# CD72 regulates the growth of KIT-mutated leukemia cell line Kasumi-1

**DOI:** 10.1038/srep02861

**Published:** 2013-10-04

**Authors:** Tatsuki R. Kataoka, Atsushi Kumanogoh, Masahiro Hirata, Koki Moriyoshi, Chiyuki Ueshima, Masahiro Kawahara, Tatsuaki Tsuruyama, Hironori Haga

**Affiliations:** 1 Department of Diagnostic Pathology, Kyoto University Hospital, Kyoto, Japan; 2 Department of Immunopathology, Research Institute for Microbial Diseases, Osaka University, Suita, Osaka, Japan; 3 WPI Immunology Frontier Research Center, Osaka University, Suita, Osaka, Japan; 4 Department of Hematology and Oncology, Kyoto University Hospital, Kyoto, Japan

## Abstract

Gain-of-function mutations in KIT, a member of the receptor type tyrosine kinases, are observed in certain neoplasms, including mast cell tumors (MCTs) and acute myelogenous leukemias (AMLs). A MCT line HMC1.2 harboring the KIT mutation was reported to express CD72, which could suppress the cell proliferation. Here, we examined the ability of CD72 to modify the growth of AMLs harboring gain-of-function KIT mutations. CD72 was expressed on the surface of the AML cell line, Kasumi-1. CD72 ligation by an agonistic antibody BU40 or by a natural ligand CD100, suppressed the proliferation of the Kasumi-1 cells and enhanced cell death, as monitored by caspase-3 cleavage. These responses were associated with the phosphorylation of CD72, the formation of the CD72 - SHP-1 complex and dephosphorylation of src family kinases and JNK. Thus, these results seemed to suggest that CD72 was the therapeutic potential for AML, as is the case of MCTs.

The receptor tyrosine kinase, KIT regulates mast cell growth differentiation and survival^1^. When the KIT ligand, stem cell factor (SCF), binds to this receptor, KIT dimerizes, autophosphorylates and consequentially activates various downstream signaling events, including src family kinases (SFKs), phosphoinositide 3-kinase (PI3K). phospholipase Cγ (PLCγ) and mitogen-activated protein kinases (MAPKs)^2^. Specific gain-of-function mutations in KIT, which induce constitutive autophosphorylation of KIT are observed in various tumors, including gastrointestinal stromal tumors (GISTs)^3^,^4^ and mast cell tumors (MCTs)^5^,^6^. The V560G mutation in the juxtamembrane domain of KIT, most frequently detected in GISTs, is sensitive to tyrosine kinase inhibitors for example imatinib^1^,^3^,^4^. However, most clinical cases of MCTs harbor the D816V mutation in the catalytic domain of KIT^5^,^6^. In contrast to GISTs, MCTs expressing D816V KIT genes are imatinib-resistant^7^,^8^. Thus, other approaches to control the mutated KIT-driven growth of MCTs are of clinical relevance^9^.

We have reported that the activation of the inhibitory molecule CD72 could inhibit the growth of HMC1.2 cells, a rapidly proliferating human mast cell line driven by V560G and D816V KIT^10^. CD72 is a transmembrane protein of the C type lectin family, which contains ITIM motifs in its cytoplasmic domain^11^. The natural ligand for CD72 was identified to be CD100/Semaphorin 4D, and specific antibodies against CD72 are known to mimic the consequences of ligation of CD72 by CD100^12^,^13^. Stimulation with CD100 or the agonistic antibodies to CD72 showed both positive and negative effects on B cell function, which may be dependent on the stage of B cell development^11^. In human mast cells, we observed that CD72 activation induced negative effects on mast cell growth and function^10^. As in B cells, negative signals mediated via CD72 in mast cells are thought to be mediated by the formation of a CD72 - SHP-1 complex^10^,^14^,^15^.

KIT mutations are also seen in patients with acute myeloid leukemias (AMLs), especially core binding factor AMLs (CBF-AMLs)^16^,^17^,^18^. CBF-AMLs are defined as AMLs with chromosomal aberrations affecting CBF transcription factor genes, such as t(8; 21) and inv(16)^19^. The former aberrations produce the fusion protein AML1-ETO, and the latter does another fusion protein CBFβ-MYH11. Studies using mouse models revealed that some mutations in tyrosine kinases are thought to be necessary for the development of CBF-AMLs, in addition to the formations of the fusion proteins^19^. The Kasumi-1 cell line provides a model for CBF-AML biology^20^. The Kasumi-1 cell line which harbors an activating mutation in KIT was established from a patient with AML M2 subtype^21^,^22^, and thus provided a model for CBF-AMLs^16^,^17^,^18^. In the case of the Kasumi-1 cell line, the N822K mutation in KIT is regarded as indispensable for the development^22^.

Here, we examined whether CD72 could inhibit the growth of Kasumi-1 cells, as was observed with HMC1.2 cells. We show that Kasumi-1 cells express CD72, and that crosslinkage of CD72 suppressed the growth of the cells by the activation of SHP-1 and the resulting down-regulation of SFKs and JNK. Thus, CD72 might present an opportunity for the targeted treatment of AMLs with KIT mutation.

## Results

### Kasumi-1 cells express CD72

**Figure 1:**
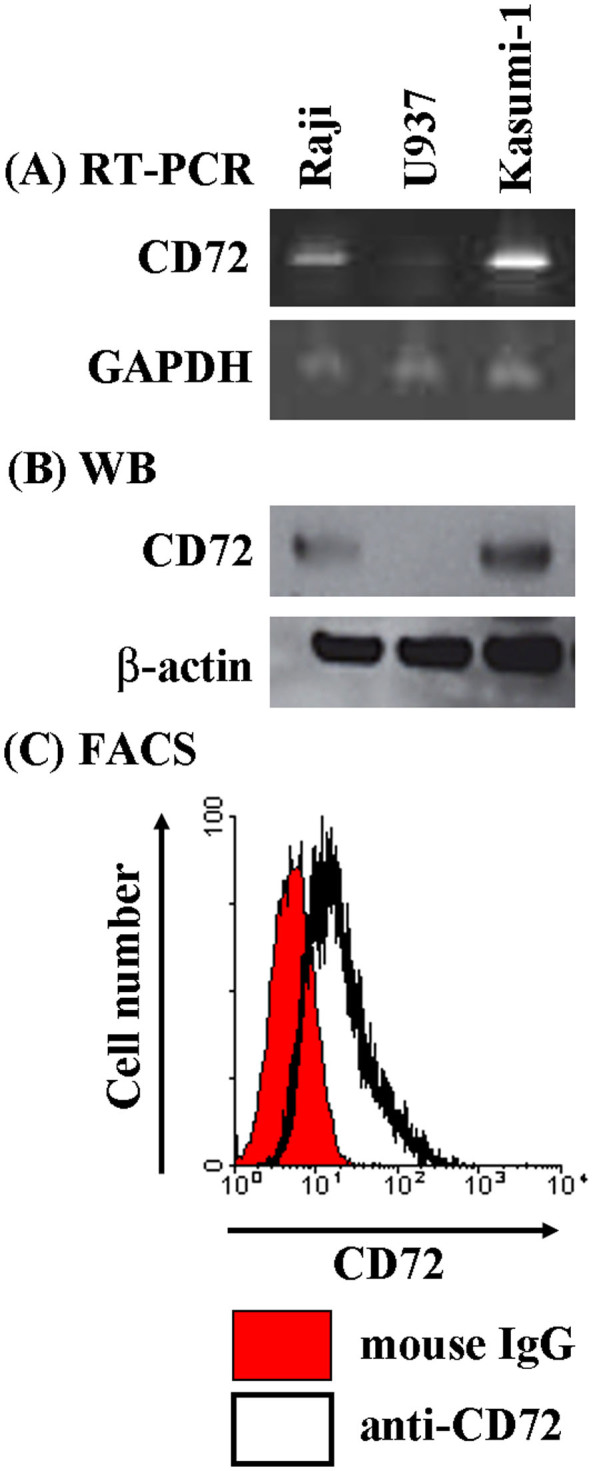
AML cell line Kasumi-1 express CD72. (A) RT-PCR. (B) Western blotting. The cropped blots were shown, and the gels have been run under the same conditions. (C) Flow cytometry.

To explore the potential ability of CD72 to regulate AML growth, we utilized the human AML cell line Kasumi-1. We first examined the expression of CD72 mRNA and protein in the cells. We executed RT-PCR to detect CD72 mRNA using two sets of primers targeting the exon1 to exon6 or exon2 to exon8 of CD72 mRNA as previously described^10^. The human Burkitt's lymphoma B cell line Raji was used as a positive control and the human monocytic cell line U937 as a negative control^23^. The expression of CD72 mRNA was detected in the Raji cells, but not in the U937 cells, as we expected (Fig. 1A). And, we detected the expression of CD72 mRNA in Kasumi-1 also (Fig. 1A). Next we executed western blotting analysis to confirm CD72 expression at protein level in the Kasumi-1 cells. This analysis showed the CD72 protein expression in the Kasumi-1 cells, like Raji cells (Fig. 1B). Additionally, we also executed FACS analysis to confirm the surface expression of CD72 on the surface of Kasumi-1 cells using an anti-CD72 antibody, BU40. This assay also revealed the expression of CD72 on the Kasumi-1 cells (Fig. 1C). Thus, we determined the expression of CD72 at mRNA and protein level in the Kasumi-1 cells.

### CD72 ligation induces the phosphorylation of CD72 and the interaction between CD72 and phospho-SHP-1 in Kasumi-1 cells

**Figure 2:**
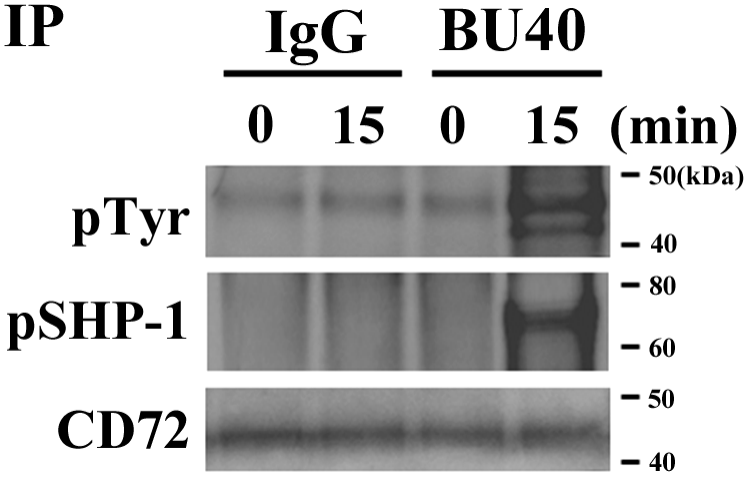
BU40 administration to Kasumi-1 cells induces the tyrosine phosphorylation of CD72and association of CD72 and phospho-SHP-1. After Kasumi-1 cells were incubated for the indicated time with control IgG or BU40, CD72 was immunoprecipitated with anti-CD72 (H-96), and visualized with anti-phospho-tyrosine, anti-phospho-SHP-1, or anti-CD72. The cropped blots were shown, and the gels have been run under the same conditions. Data are representative from three individual experiments.

In HMC1.2 cells, we observed that CD72 ligation by BU40 induced the phosphorylation of CD72 and the formation of a CD72 – SHP-1 complex with resulting decrease in the phosphorylation of specific signaling molecules^10^. Therefore, we examined whether CD72 was phosphorylated resulting in formation of a CD72-SHP-1 complex in the Kasumi-1 cells, as is the case of HMC1.2. As shown in Fig. 2, immunoprecipiation assay revealed the phosphorylation of CD72. SHP-1 is known to be phosphorylated and activated its dephosphorylation ability^24^. We thus examined the status of SHP-1 binding to CD72 in Kasumi-1. In the assay, we observed that phosphorylated SHP-1 bound to CD72 by BU40 administration (Fig. 2).

### CD72 ligation suppresses the proliferation of Kasumi-1 cells

**Figure 3:**
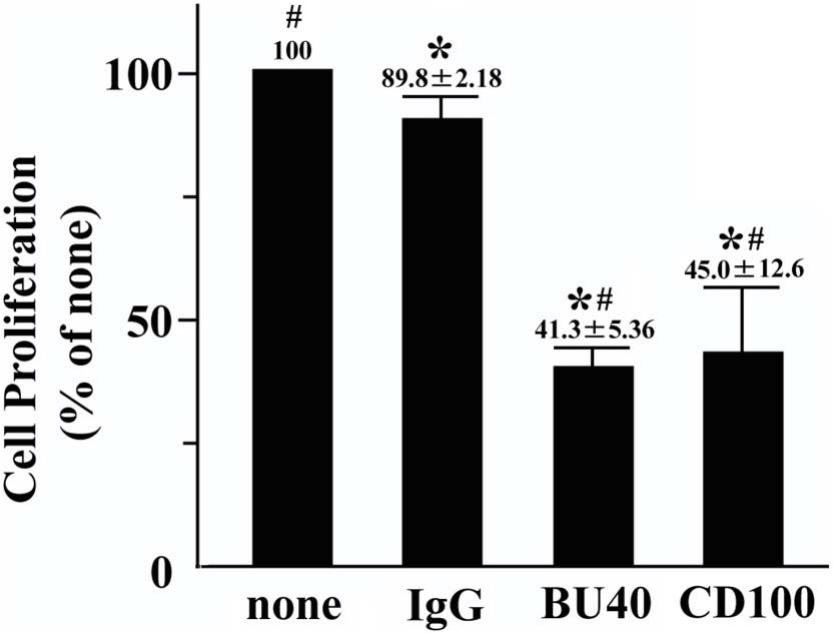
The stimulation on CD72 with BU40 suppresses cell proliferation of Kasumi-1 (Brd U assay, n = 4). Kasumi-1 cells were incubated for 24 hours with vehicle, control IgG, BU40, or recombinant CD100. *; *P* < 0.01, when compared with the value of vehicle. #; *P* < 0.01, when compared with the value of control IgG.

We next evaluated the effect of CD72 ligation on the proliferation of Kasumi-1 cells. The anti-CD72 antibody BU40 or recombinant CD100 (rCD100) was used for this purpose, as previously described^12^,^25^. The proliferation was monitored by BrdU incorporation into DNA. The administration of control IgG did not significantly affected the proliferation of the Kasumi-1 cells (Fig. 3). In contrast CD72 ligation by either BU40 or rCD100 markedly reduced the proliferation of Kasumi-1 cells (Fig. 3).

### CD72 ligation enhances the cell death of Kasumi-1 cells

**Figure 4:**
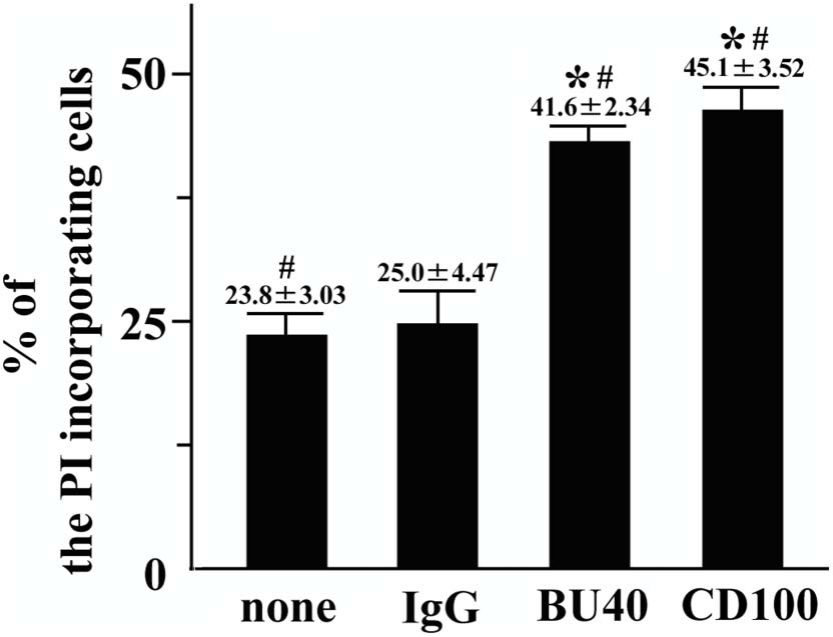
BU40 induces the cell death of Kasumi-1. Kasumi-1 cells cultured in HEPES + 0.04% BSA with vehicle, control IgG, BU40 or recombinant CD100 for 24 hours. The cells stained with propidium iodine (PI) without fixation, then analyzed using a FACScan flow cytometer. The cells incorporating PI were considered as the dead cells. *; *P* < 0.01, when compared with the value of vehicle. #; *P* < 0.01, when compared with the value of control IgG.

CD72 regulates B cell death both positively^26^ and negatively^27^. Thus, we assessed whether the CD72 ligation enhanced or inhibited the Kasumi-1 cell death. We determined the dead cells as PI-incorporating cells by the FACS analysis^28^. Isotype control antibody administration did not influence the death of Kasumi-1 cells (Fig. 4). However, CD72 ligation induced by both BU40 and rCD100 significantly increased the proportion of the dead Kasumi-1 cells (Fig. 4).

### CD72 ligation induces down-regulation of the phosphorylation of signal molecules in Kasumi-1 cells

**Figure 5:**
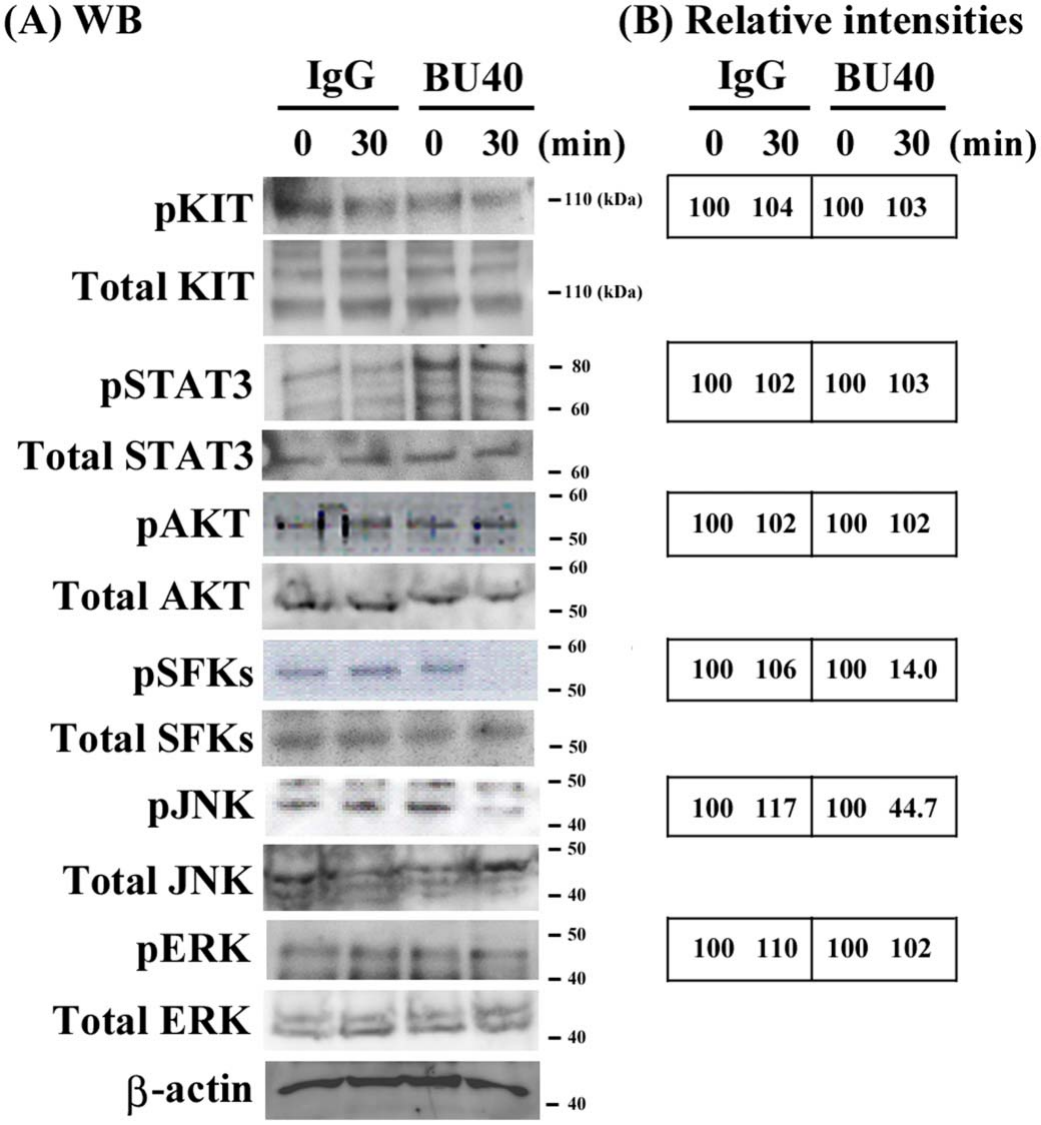
BU40 administration to Kasumi-1 cells suppresses the activation of signal molecules. Kasumi-1 cells were incubated for the indicated time with control IgG or BU40. The levels of phospho-KIT, KIT, phospho-STAT3, STAT3, phospho-AKT, AKT, phospho-Src family kinases (Tyr 416), non-phospho-Src family kinases, phospho-JNK, JNK, phospho-ERK, ERK, and β-actin were evaluated. (A) Data are representative from three individual experiments. Data are representative from three individual experiments. The cropped blots were shown, and the gels have been run under the same conditions. (B) The intensities of the bands were normalized to the intensities of the corresponding bands for β-actin. The relative values of phospho-KIT, phospho-STAT3, phospho-AKT, phospho-Src family kinases (Tyr 416), phospho-JNK, phospho-ERK, at 15 min with IgG or BU40 were calculated when those at 0 min were 100.

To investigate the mechanisms that underlie the suppressed proliferation of Kasumi-1 cells by CD72 ligation, we explored the changes in the status of signal molecules following the addition of the ligating CD72 Ab. PI3K, as indicated by AKT phosphorylation, JNK, and STAT3 were reported to be constitutively activated in KIT-mutated cell line Kasumi-1^29^. Additionally, SFKs and ERK are activated by KIT activation^2^. Therefore, we examined the status of these signaling molecules after treating the cells with control IgG or BU40 for 30 min. As shown in Fig. 3A the phosphorylation of JNK and SFKs were significantly reduced in the cells treated with BU40 (Fig. 5). However, there were no marked differences in the phosphorylation of ERK, AKT and STAT3 (Fig. 5) following CD72 ligation with BU40.

### CD72 ligation induces the cleavage of caspase3 in Kasumi-1 cells

**Figure 6:**
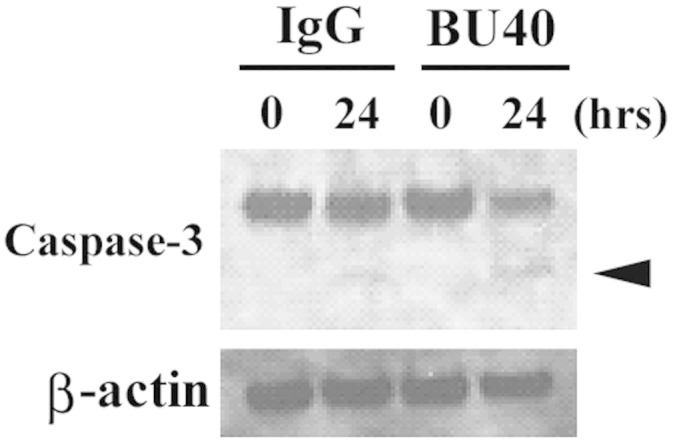
BU40 administration to Kasumi-1 cells enhances the cleavage of caspase-3. The levels of caspase-3 and β-actin were evaluated. The band which arrowhead indicated the size corresponding to cleaved caspase-3. Data are representative from three individual experiments. The cropped blots were shown, and the gels have been run under the same conditions.

Next, we explored the molecular basis of the increased cell death by CD72 ligation. Increased cleavage of caspase-3 in Kasumi-1 was observed when certain drugs induced the cell death of Kasumi-1^30^,^31^,^32^,^33^. Therefore, we examined the status of caspase-3 after 12 h or 24 h BU40 administration by western blotting. Non-cleaved caspase-3 was decreased, and at the same time cleaved forms of caspase-3 was increased by BU40 administration, when compared to the control (Fig. 6). When BU40 was added, cleaved caspase-3 was confirmed after 24 h administration, though control IgG induced undetectable level of the cleavage of caspase-3 (Fig. 6).

## Discussion

Gain-of-function mutations in the KIT play central roles in the development of certain tumors, including MCTs and AMLs^1^. Both a MCT line HMC1.2 and an AML line Kasumi-1 harbor mutations in different sites of KIT; V560G and D816V in HMC1.2, and N822K in Kasumi-1^5^,^22^. There is a difference between the biological characteristics of HMC1.2 and that of Kasumi-1, in addition to the sites of KIT mutations. For example, HMC1.2 cells were imatinib-resistant^7^,^8^, although Kasumi-1 cells were sensitive to imatinib^29^. The current study also showed an additional difference between the characteristics of these two lines; CD72 ligation decreased the phosphorylation of KIT at the codon 703 in HMC1.2, but did not in Kasumi-1. This difference would be due to the sites of KIT mutation or to the presence or absence of AML1-ETO^5^,^22^. The phosphorylation of the codon 703 of KIT activates Grb2/RAS/RAF signal pathway^35^. Grb2/RAS/RAF is located at the upstream of ERK^2^, and CD72 ligation-resistant ERK phosphorylation in Kasumi-1 cells might be explained by persistent phosphorylation of KIT at codon 703.

Nevertheless, HMC1.2 and Kasumi-1 have common phenotype on CD72; CD72 could suppress the proliferation of Kasumi-1 (Fig. 3), as is the case of HMC1.2^10^. The mechanism of the suppressed proliferation by CD72 ligation in both cell lines was common as followed; CD72 ligation induced the phosphorylation of CD72 itself, and the formation of the CD72 – SHP-1 complex, perhaps resulting in the down-regulation of activations of SFKs and MAPKs. Both signal molecules are thought to play an important role in the proliferation of AML cells^30^,^35^,^36^. Therefore, it appears that the down-regulation of SFKs and JNKs mediated the reduced proliferation of Kasumi-1 by CD72 ligation.

The AKT phosphorylation was not influenced by CD72 ligation in either Kasumi-1 (Fig. 3) or HMC1.2^10^. We thought that this would be because PI3K – AKT could not be suppressed by SHP-1, activated by the CD72 ligation in Kasumi-1 and HMC1.2. CD72 ligation partially, not fully, suppressed the proliferation of Kasumi-1 (Fig. 2) or HMC1.2^10^, partially. AKT would maintain the CD72 ligation-resistant proliferative activities of these cells.

In the current study, we observed CD72 ligation enhanced the cell death of Kasumi-1 and the cleavage of caspase-3 (Fig. 4 & 6). The increased cleavage of caspase-3 has been reported to be associated to Kasumi-1 cell death following addition of certain inhibitors^30^,^31^,^32^,^33^,^34^. Therefore, we concluded that CD72 induced the cleavage of caspase-3 and ended up with Kasumi-1 cell death, as is the case of the inhibitors. Here, we also observed the suppression of JNK and SFK activation by the CD72 ligation (Fig. 3). There are reports that the JNK inhibitor SP600125 enhanced the cleavage of caspase-3 and apoptosis of Kasumi-1 cells^34^, and that PP2, an inhibitor of SFKs, also induced the cell death of Kasumi-1 cells^33^. Then, we supposed that the down-regulation of JNK and SFK activation by CD72 ligation was involved in the cleavage of caspase-3 and the followed cell death of Kasumi-1.

Some antibodies are proposed as a tool for the treatment for AMLs. Among them, anti-CD33 antibody has been well-studied and established^35^. The suppressive effect of anti-CD33 antibody on the growth of AML was mediated by SHP-1^36^, as is the case of BU40. Our current study seems to suggest that anti-CD72 antibody BU40 may provide a novel approach for the treatment of AMLs via SHP-1.

## Methods

### Cells

We used Kasumi-1, Raji, and U937 cells for this study. All cell lines were obtained from American Type Culture Collection (Manassas, VA). Kasumi-1 cells were grown in RPMI1640 medium containing 20% FBS, _L_-glutamine (2 mM), penicillin (100 units/ml), and streptomycin (100 μg/ml) (Invitrogen, Calrlsbad, CA). Raji and U937 cells were grown in RPMI1640 containing 10% FBS, _L_-glutamine (2 mM), penicillin (100 units/ml), and streptomycin (100 μg/ml) (Invitrogen).

### Recombinant CD100 and antibodies

Recombinant CD100 protein was prepared as described^25^. Anti-human CD72 antibody (Ab) (Clone; BU40, monoclonal, mouse IgG) was purchased from Southern Biotechnology Associates (Birmingham, AL) for the stimulation on Kasumi-1 cells or the flow cytometry. Another anti-CD72 Ab (H-96, rabbit polyclonal IgG) for the immunoprecipitaion assay, anti-SHP-1 Ab (C-19, rabbit polyclonal IgG), and anti-KIT Ab (C-19, rabbit polyclonal IgG) were purchased from Santa Cruz Biotechnology (Santa Cruz, CA). Anti-phosphotyrosine Ab (4G10, mouse monoclonal IgG) was purchased from Millipore (Billerica, MA), and anti-phospho-KIT (Tyr 703) Ab (rabbit polyclonal IgG) was purchased from Invitrogen. Anti-phospho-Src Ab (Tyr 416), anti-non-phospho-Src Ab (Tyr 416), anti-phospho-STAT3 Ab (Tyr 705), anti-STAT3 Ab, anti-phospho-AKT Ab (Thr 308), anti-AKT Ab, anti-phospho-JNK Ab (Thr 183/Tyr 185), anti- JNK Ab, anti-phospho-ERK Ab (Thr 202/Tyr 204) and anti-phospho-ERK Ab (these Abs were rabbit polyclonal IgG) were obtained from Cell Signaling Technology (Beverly, MA). Anti-phospho-SHP-1 (Tyr 536, rabbit polyclonal IgG) was obtained from ECM Biosciences (Versailles, KY). Anti-β-actin Ab (mouse monoclonal IgG) was obtained from Sigma-Aldrich (St. Louis, MO). Isotype control Abs were obtained from BD Biosciences (San Jose, CA). The secondary Abs were peroxidase-labeled anti-rabbit or anti-mouse IgG antibodies (Santa Cruz Biotechnology).

### RT-PCR

Five × 10^6^ cells (Raji, U937, or Kasumi-1) was collected by centrifugation and processed with TRIzol (Invitrogen). We extracted total RNAs using RNeasy columns according to the manufacturer's instructions (Invitrogen). One μg of each RNA sample was used for RT-PCRs (SuperScript III One-Step RT-PCR System; Invitrogen). We prepared the same sets of primers as followed^10^; exon1 (5′-ATGGCTGAGGCCATCACCTA-3′), exon2 (5′-ACACCTGCTGTCCGTCG-3′), exon5 (5′-CTGCTGAGCCGCATGTG-3′), and exon8 (5′-CTAATCTGGAAACCTGAAAGCTG-3′). cDNA synthesis and PCR amplification were performed with a DNA Engine PTC-200 cycler (Bio-Rad Laboratories) as followed; cDNA synthesis: 30 min at 55°C; denaturation: 2 min at 94°C; PCR amplification (30 cycles): 30 sec at 94°C, 1 min at 60°C; 1 min at 72°C; final extension at 10 min at 72°C^10^.

### Flow cytometry

We analyzed the surface expression of CD72 on Kasumi-1 cells using a FACScan flow cytometer. Kasumi-1 cells were washed, fixed with 4% paraformaldehyde (Sigma-Aldrich), and stained with anti-human CD72 (BU40) for overnight at 4°C. After washing, anti-mouse IgG2a-FITC was added and reacted to the cells for 2 hours at 4°C. The cells were then analyzed.

### Bromodeoxyuridine (BrdU) cell proliferation assay

Kasumi-1 cells were cultured overnight in cytokine-free medium. After washing by cytokine-free medium, the cells re-cultured for 24 h at a density of 1.5 × 10^5^ cells/100 μl in 96-well plates in RPMI1640 containing 10% FCS with or without control IgG, recombinant CD100, or BU40 (10 μg/ml, dissolved in PBS + 0.04% NaN_3_, respectively). Cell proliferation was assessed using a BrdU cell proliferation assay kit (Calbiochem, San Diego, CA) as previously described^37^.

### Cell death

We analyzed the Kasumi-1 cell death as described^28^. Kasumi-1 cells were cultured overnight in cytokine-free medium, and resuspended in HEPES buffer containing 0.04% BSA. The cells were cultured for additional 24 h with or without control IgG, recombinant CD100, or BU40 (10 μg/ml, dissolved in PBS + 0.04% NaN_3_, respectively). The cells were stained with propidium iodine (Sigma-Aldrich) without fixation for 2 h at 4°C, and analyzed using a FACScan flow cytometer. The cells incorporating PI were considered as dead cells.

### Immunoblotting and immunoprecipitation

Cell lysates were prepared, the proteins separated by electrophoresis, and gels probed for immunoreactive proteins as described^10^. Immunoprecipitation experiments were executed using an anti-CD72 Ab (H-96) as described^10^. The cells were lysed in buffer containing 150 mM NaCl, 10 mM Tris-HCl (pH 8.0), 1 mM EDTA, 1 mM Na_3_VO_4_, 0.5 mM PMSF, 5 μg/ml aprotinin, 5 μg/ml leupeptin, complete protease inhibitor cocktail (Roche, Indianapolis, IN), and NP-40 (1%). The cell lysates were incubated with rabbit IgG-bound protein G-sepharose for 30 min, then incubated with anti-CD72 Ab (H-96)-bound protein G-sepharose overnight at 4°C with rotation. Immunoprecipitated protein samples were probed by anti-phospho-tyrosine Ab, anti-phospho-SHP-1, or anti-CD72 after blotting. The blotting images were scanned using a Light-Capture II (ATTO, Tokyo, Japan). To quantitate the bands' intensities, the scanned data was analyzed by CS Analyzer (ATTO).

### Statistical analysis

Data were expressed as the means ± SE. Differences between groups were examined for statistical significance using Student's *t*-test (Excel: Microsoft, Redmond, WA, USA). A *P* value less than 0.05 indicated statistical significance.

## Author Contributions

T.R.K. prepared figures 1–6, wrote the manuscript and reviewed this study. A.K. prepared the antibody and the recombinant protein. M.H., K.M. and M.K. prepared figures 5, 6. C.U. prepared figures 5 and the revised work, and reviewed the manuscript. T.T. and H.H. wrote the main manuscript text, and reviewed this study. All authors reviewed the manuscript.

## Supplementary Material

Supplementary Information Fig2-Fig5 suppl
