# Supplementary material for: CD72 regulates the growth of KIT-mutated leukemia cell line Kasumi-1
Source: Sci Rep. 2013 Oct 4;3:2861. doi: 10.1038/srep02861 (PMC3980566; doi:10.1038/srep02861)

**SREP-13-01610B**

CD72 regulates the growth of KIT-mutated leukemia cell line Kasumi-1

Tatsuki R. Kataoka, Atsushi Kumanogoh, Masahiro Hirata, Koki Moriyoshi, Chiyuki Ueshima, Masahiro Kawahara, Tatsuaki Tsuruyama, Hironori Haga.

**Figure 2 suppl files**

1) pTyr


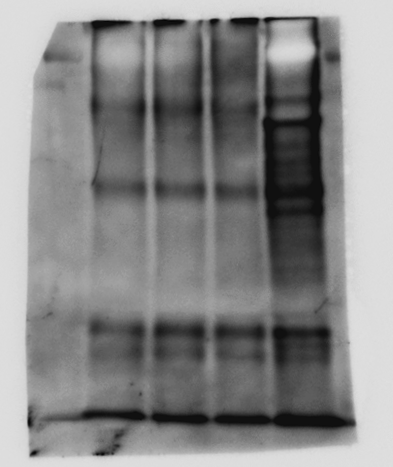


2) pSHP-1


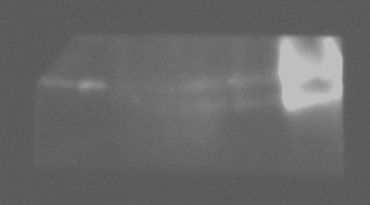


3) CD72


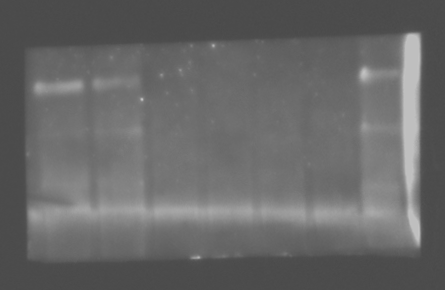


**Figure 5 suppl files**

1) pKIT


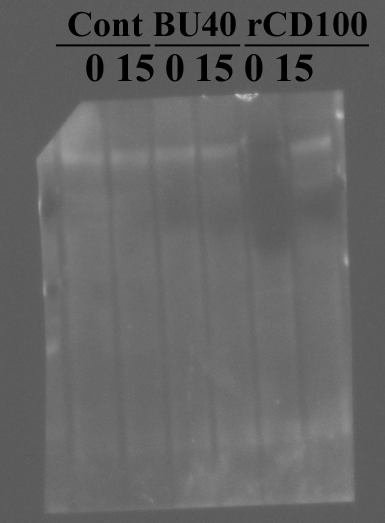


2) Total KIT


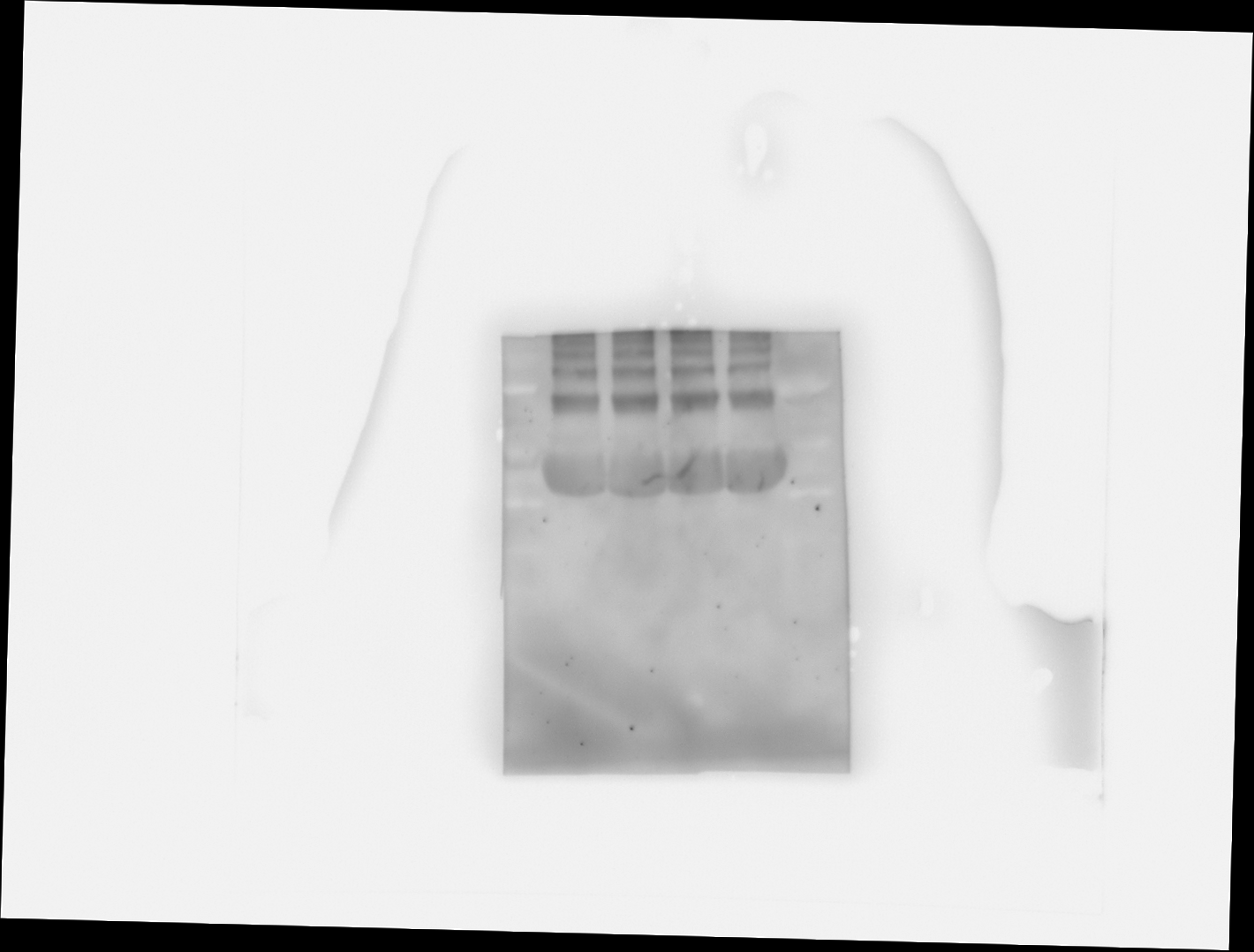


3) pSTAT3


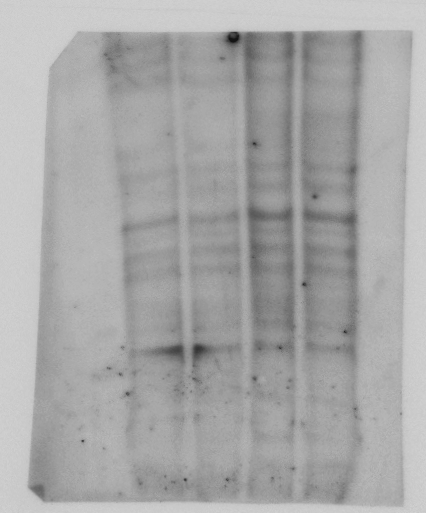


4) Total STAT3


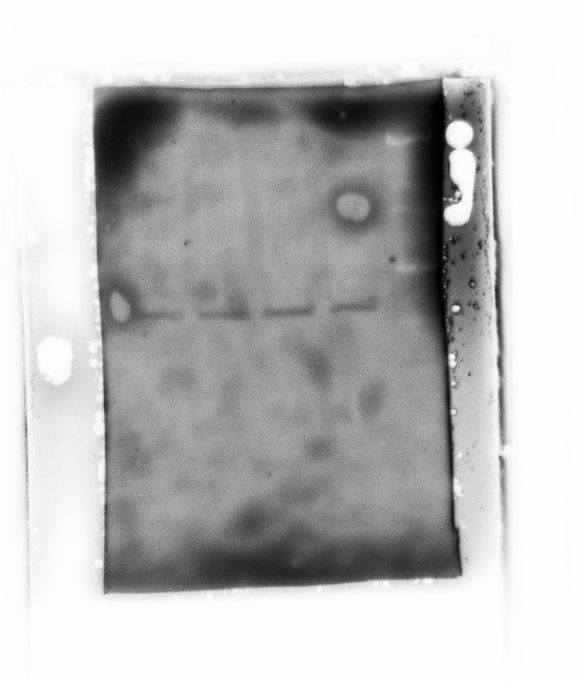


5) pAKT


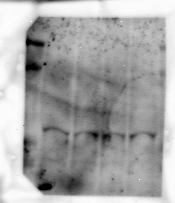


6) Total AKT


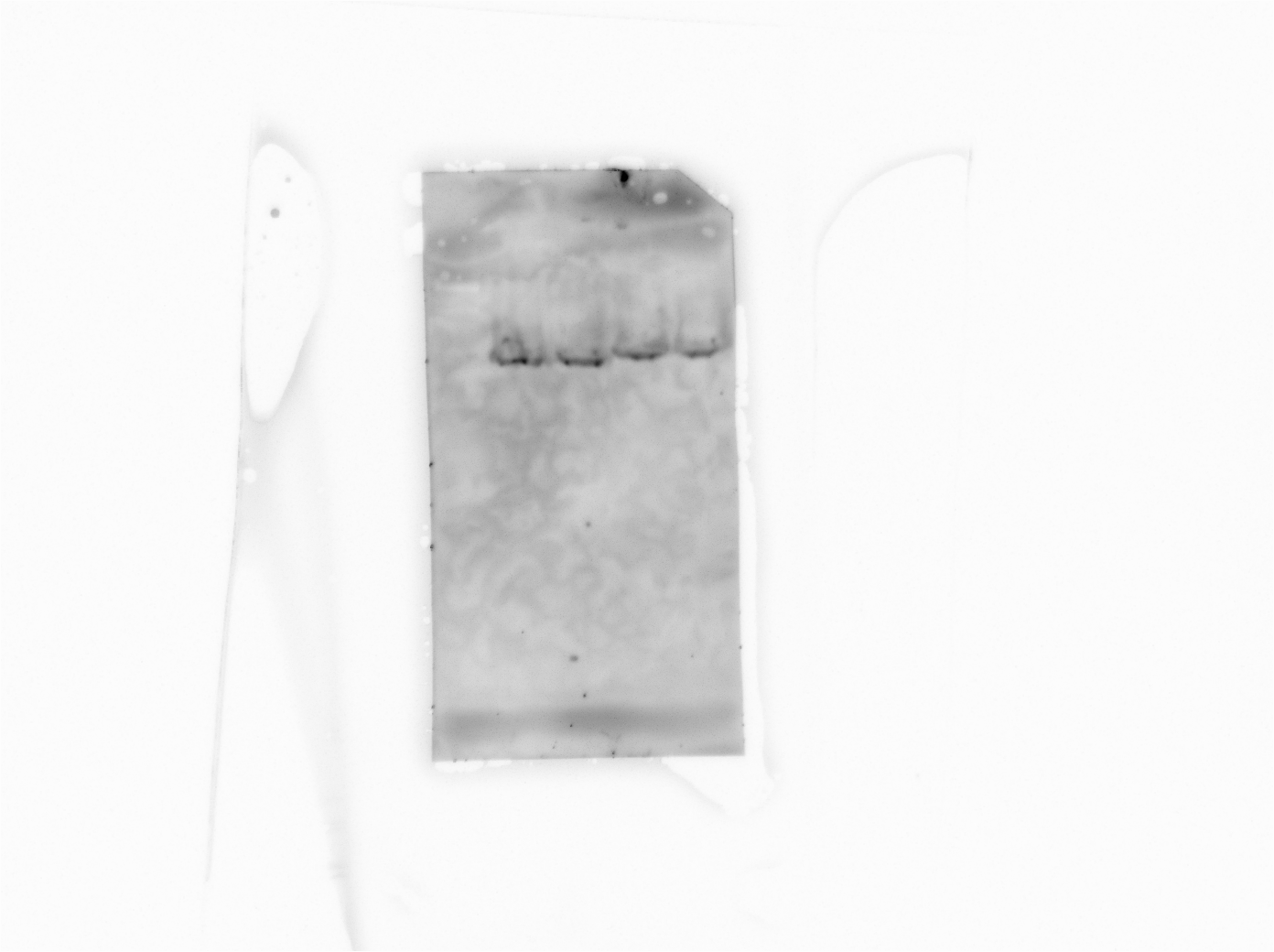


7) 9) pSFK & pJNK


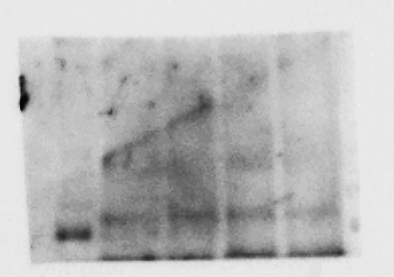


8) Total SFK


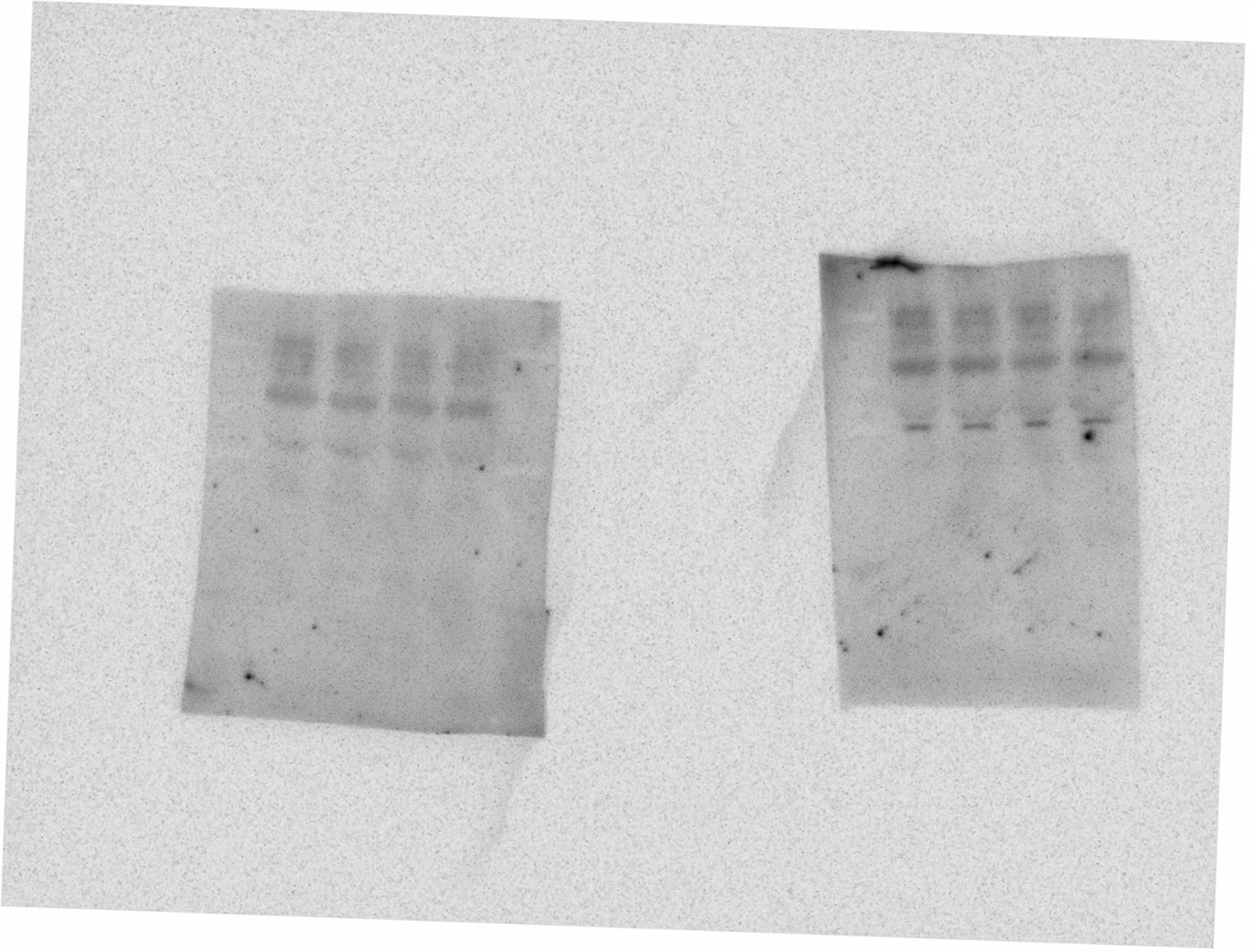


10) Total JNK


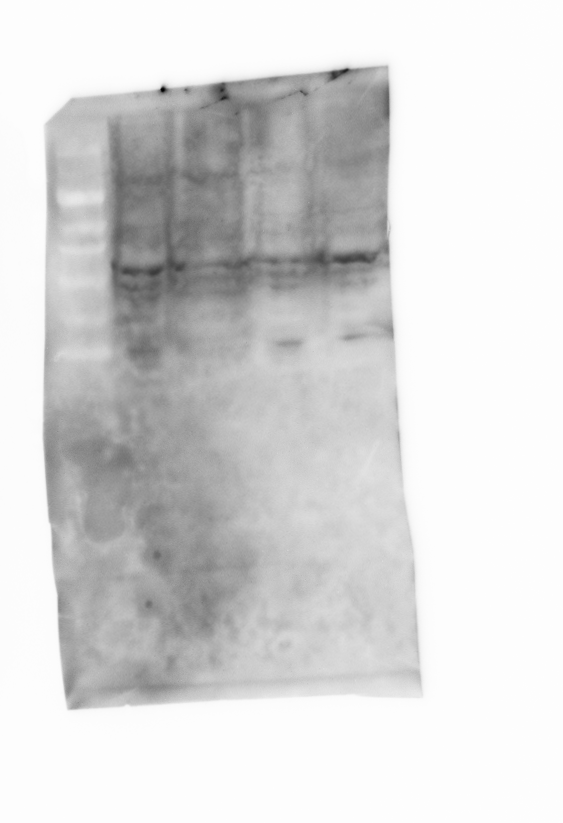


11) pERK


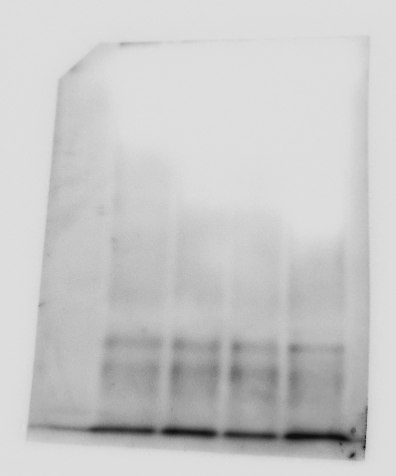


12) ERK


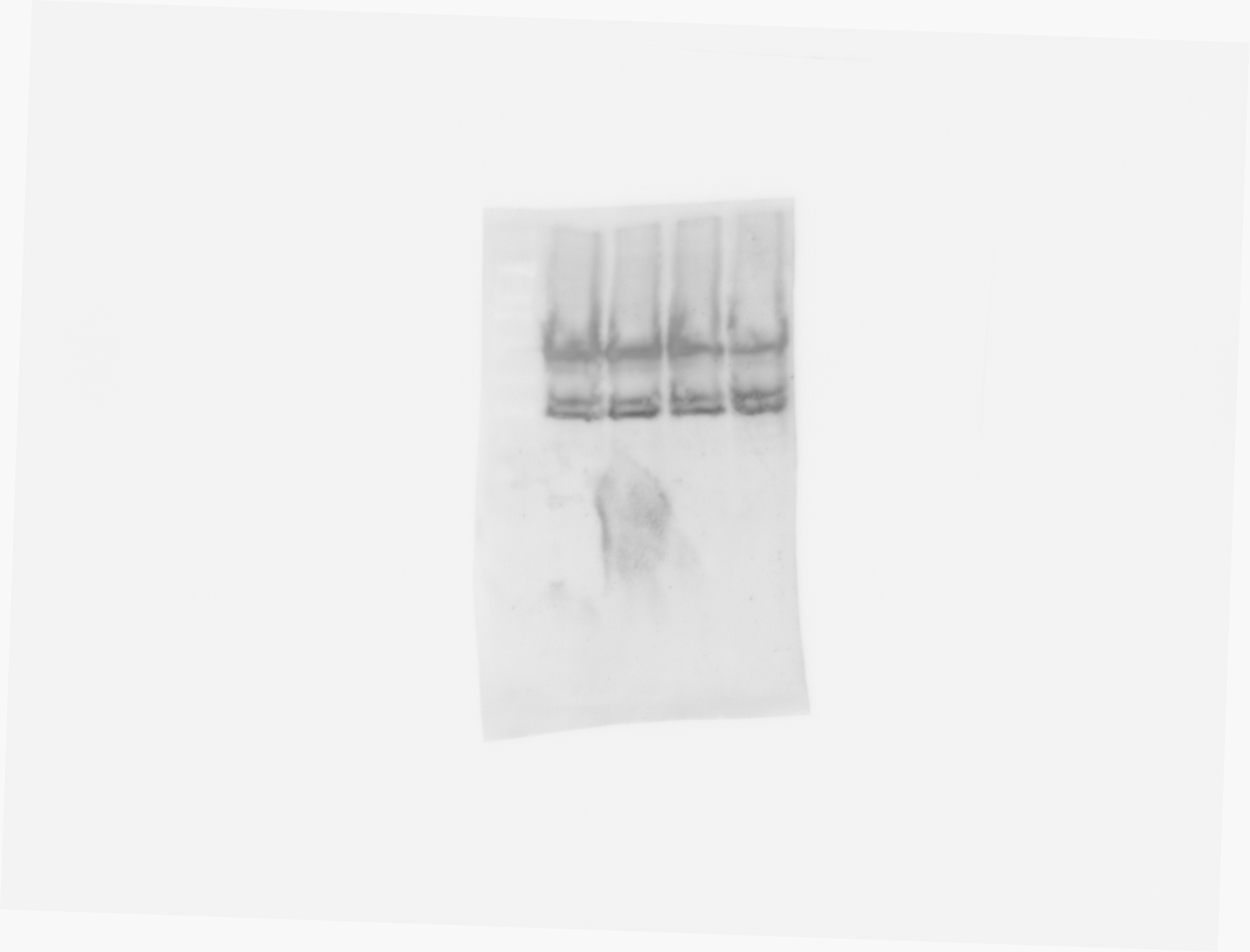


13) beta-actin


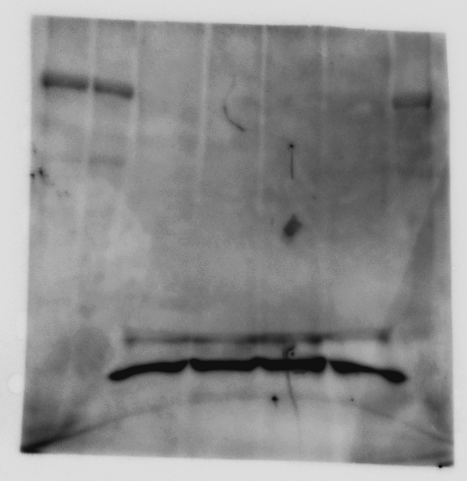

Supplement: Supplementary Information — Fig2-Fig5 suppl [file srep02861-s1.doc]
